# The Testing of a Four-Dimensional Model of Athlete Leadership and Its Relation to Leadership Effectiveness

**DOI:** 10.3389/fpsyg.2020.01361

**Published:** 2020-07-03

**Authors:** Christopher Maechel, Todd M. Loughead, Jürgen Beckmann

**Affiliations:** ^1^Chair of Sport Psychology, Department of Sport and Health Sciences, Technical University of Munich, Munich, Germany; ^2^Sport and Exercise Psychology Laboratory, Department of Kinesiology, University of Windsor, Windsor, ON, Canada; ^3^School of Human Movement and Nutrition Sciences, The University of Queensland, Brisbane, QLD, Australia

**Keywords:** athlete leadership, shared leadership, functional leadership theory, change-oriented leadership, leadership effectiveness

## Abstract

Athlete leadership researchers have typically investigated three dimensions of athlete leadership behaviors, which include the meta-categories of task-, social-, and external-oriented leadership. More recently, motivational leadership was added as a fourth dimension. Researchers in organizational leadership have advanced another dimension, referred to as change-oriented leadership (Yukl, 2012). Therefore, in the present study, we tested a four-dimensional model that includes the dimensions of task-, social-, external-, and change-oriented leadership. Two samples of 161 athletes and 69 coaches rated every player on their team on the four-dimensional model and on perceived athlete leadership effectiveness. A multilevel regression analysis showed that all four dimensions of athlete leadership significantly predicted perceived athlete leadership effectiveness for players and three dimensions (i.e., social-, task-, and change-oriented leadership) for coaches. These results support the importance of change-oriented leadership in relation to athlete leadership.

## Introduction

Leadership is a crucial component for team functioning in high-performance sport teams (Chelladurai, 2007). For instance, researchers have demonstrated that effective leadership is associated with increased individual performance (Bormann and Rowold, 2016), positive motivational climate (Seifriz et al., 1992; Duda, 2001), intrinsic motivation (Amorose and Horn, 2000), collective efficacy (Magyar et al., 2004; Price and Weiss, 2013), increased team cohesion, and athlete satisfaction (Kim and Cruz, 2016). These results are not surprising since leadership constitutes a fundamental process in group dynamics. However, research has predominantly focused on individualistic, top-down forms of leadership (i.e., coaches, managers), mostly disregarding lateral or bottom-up leadership (i.e., athletes). In the last decade, the study of athlete leadership has emerged as a research area, investigating leadership provided by the athletes to their teams. In fact, empirical studies have demonstrated a relationship between athlete leadership and team cohesion (Price and Weiss, 2011; Loughead et al., 2016), team resilience (Morgan et al., 2013, 2015), athlete satisfaction (Eys et al., 2007), role clarity (Crozier et al., 2013), and team effectiveness (Fransen et al., 2017).

By definition, athlete leadership is viewed as athletes occupying a formal or informal leadership role within the team and influencing team members to achieve a common goal (Loughead et al., 2006) – that is, athletes emerge as leaders by fulfilling either a formal or an informal leadership role. The former refers to those players who are designated with an official leadership role, such as captain or assistant captain. The latter refers to those athletes who emerge as leaders as a result of social interaction and are regarded by their teammates as providing leadership. By having both formal and informal leaders fulfill leadership roles, the definition implicitly acknowledges that athlete leadership on sport teams is a shared process, which is investigated in the shared leadership literature (Pearce and Conger, 2003). A key tenet of shared leadership is that the complexity and the ambiguity make it difficult for a single leader to successfully perform all the various leadership functions. In other words, “leadership is probably best conceived as a group quality, as a set of functions which must be carried out by the group” (Gibb, 1954, 884). This shared element is also captured by other leadership theories, such as functional leadership theory (McGrath, 1962; Morgeson et al., 2010). This theory suggests that leaders need “to do, or get done, whatever is not being adequately handled for group needs” (McGrath, 1962, 5). This implies that the leadership functions, which serve to meet the team’s needs, do not need to be performed by the same individual; rather, anyone who fulfills these responsibilities is considered to assume a leadership role. As such, Loughead et al. (2019) noted the shared nature of athlete leadership by indicating that it is “a shared team process comprised of mutual influence and shared responsibility amongst team members, who lead each other toward the achievement of a common goal.”

The shared nature of athlete leadership has been demonstrated in several studies using various research methodologies. For instance, Loughead et al. (2006) used dispersion statistics to highlight the shared nature of athlete leadership. The results indicated that 8–15% of athletes were viewed as formal leaders and 29–47% of athletes were viewed as informal leaders within their respective teams. Furthermore, when team members were asked about the ideal number of athlete leaders in a team, the results showed that 85% of athletes should fulfill a leadership role (Crozier et al., 2013). Another research method used to demonstrate the shared nature of athlete leadership is social network analysis. Social network analysis is a methodological tool that examines the “relationships among social entities, and on the patterns and implications of these relationships” (Wasserman and Faust, 1994, 3). For instance, in order to visually and quantitatively examine the distribution of athlete leadership, Duguay et al. (2019b) sampled four competitive youth teams. Within each team, every player was asked to rate the extent that they looked for leadership to each of their teammates. The results showed that there were no leadership isolates, indicating that every team member provided leadership to at least one other member of the team, supporting the notion that athlete leadership is a shared phenomenon.

Given that numerous athletes are able to contribute to the leadership of the team, the question then becomes: what are the specific leadership functions that are shared? To date, athlete leadership research has focused on four leadership functions: task, social, external, and motivational. The task-related functions are oriented toward the team’s task goals (e.g., clarifying team goals) and were first identified in the Ohio State studies (Fleishman, 1953), referring to the behavioral factor of *initiating structure*, which constitutes a leaders effort toward goal attainment and the establishment of means of communication. The social-related functions are oriented toward individual team members (e.g., satisfying individual needs) and were also first identified in the Ohio State studies (Fleishman, 1953), within the behavioral factor of *consideration.* It refers to behavior oriented toward followers that demonstrate concern, appreciation, and respect as well as providing support. The external-related functions originate from research on *boundary spanning*, which can be described as an effort to initiate and manage external connections (Ancona and Caldwell, 1992; Marrone, 2010). Generally, these function to provide a team with linkages to its external environment (e.g., advocating and representing the team). Lastly, the operationalization of motivational leadership originated within athlete leadership research. Its function is to encourage teammates and promote emotions conducive to team performance during on-field situations (Fransen et al., 2014). Taken together, all four functions have shown to be empirically relevant for athlete leadership in sports. The dimensions of task and social leadership were among the first functions identified in the sports context (Rees and Segal, 1984). Later, Loughead et al. (2006) corroborated their findings while demonstrating the relevance of external leadership for athlete leadership. Lastly, Fransen et al. (2014) demonstrated that motivational leadership was present within the sports context. All four functions are used in athlete leadership research today (Cotterill and Fransen, 2016).

While the research showing the presence of the four leadership functions (task, social, external, and motivational) has helped to advance our understanding of athlete leadership, no attempt, to our knowledge, has been made to bring together these related functions in order to give a broader understanding of the phenomenon of interest, in this case, athlete leadership. Yukl (2012) advanced a taxonomy of leadership that appears to be suitable for the study of athlete leadership. Specifically, Yukl et al. (2002) reviewed 50 years of leadership research in organizational psychology, providing the most comprehensive and integrative overview of behavioral leadership research to date (Yukl et al., 2002; Yukl, 2012). They concluded that *task-oriented*, *relations-oriented*, *change-oriented*, and *external* leadership serve as the four meta-categories of effective leadership behavior, and within those four categories, a total of 15 sub-dimensions are contained. The significance for athlete leadership is twofold. First, Yukl’s taxonomy promotes conceptual clarity with regard to the relevance and the structure of leadership functions. As a research area grows, such as athlete leadership, there is usually a proliferation of taxonomies (Fleishman et al., 1991; Yukl et al., 2002). In athlete leadership research, the original three-dimensional model advanced by Loughead et al. (2006) has already been extended with the inclusion of *motivational leadership* (Fransen et al., 2014). While this fourth component has shown to be empirically relevant (Fransen et al., 2017), it does not have a comparably strong historical background as task-, social-, and external-oriented leadership (Loughead et al., 2006). In this regard, Yukl’s taxonomy provides an empirically tested and comprehensive reference point that could help to structure the existing research knowledge. For instance, *motivational leadership* shares aspects of change orientation (e.g., inspirational motivation) while disregarding others (e.g., advocating change) from Yukl’s taxonomy. On the one hand, this supports the existence and the necessity of such a leadership function. On the other hand, it raises the question on whether a four-dimensional model of athlete leadership, including task-oriented, social-oriented, external-oriented, and motivational leadership, covers all aspects of athlete leadership. Second, Yukl’s taxonomy highlights potential areas of future research. In relation to athlete leadership, change-oriented leadership has only been examined in the context of transformational leadership research (Callow et al., 2009). While there is some conceptual overlap, transformational leadership does not cover all leadership behaviors identified by the meta-category of change orientation (Yukl et al., 2002). Specifically, this meta-category refers to activities that serve to advocate for change, articulate an inspiring vision, encourage innovation, and inspire collective learning (Yukl, 2012). The importance of the change-oriented dimension is supported by various leadership theories, such as transformational or charismatic leadership (e.g., Bass, 1985; Shamir et al., 1993). Additionally, the ability to encourage innovation and provide inspiration to others has been identified as an essential component of leadership in organizational research (Williams and Foti, 2011; Waite, 2014) as well as in sport, for both coaches (Vella et al., 2012; Bormann and Rowold, 2016) and players (Callow et al., 2009). Furthermore, all four meta-categories were shown to be valid dimensions for shared leadership in organizational teams (Grille and Kauffeld, 2015).

Thus, the aim of the current study is to investigate athlete leadership using Yukl’s (2012) four meta-category taxonomy (task-oriented, relations-oriented, change-oriented, and external leadership). To accomplish this objective, the present study examined the presence of these four meta-categories, in relation to athlete leadership, by surveying both athletes and coaches. In order to evaluate the significance of the four functions of leadership, we chose to use perceived leadership effectiveness as the dependent variable. It has been shown that evaluations of leadership effectiveness correspond with objective measures of group performance (Hogan et al., 1984) and sport team performance (Fransen et al., 2017). In order to determine whether the addition of change-oriented leadership is relevant for athlete leadership research, we investigated whether this dimension contributes unique variance to a model predicting perceived leadership effectiveness and whether the inclusion of change orientation improved the model fit. Therefore, the following hypotheses were tested: for athletes (H1a) and coaches (H1b), controlling for the dimensions of social-, task-, and external-oriented leadership, change-oriented leadership will significantly predict perceived athlete leadership effectiveness. For athletes (H2a) and coaches (H2b), the four-dimensional model will show a significantly better model fit than the three-dimensional model.

## Materials and Methods

### Participants

The participants consisted of both athletes and coaches. The athletes were 161 (82 females, 79 males) German professional-level (*n* = 57), national-level (*n* = 17), regional-level (*n* = 20), and district-level (*n* = 67) athletes with an average age of 23.98 years (SD = 6.94). These athletes represented 81 different teams, from 60 clubs, and competed in a variety of interactive team sports, including volleyball (*n* = 70), basketball (*n* = 35), handball *(n* = 27), field hockey (*n* = 15), ice hockey (*n* = 7), soccer (*n* = 5), and lacrosse (*n* = 2). The coaches were 63 (57 males, six females) German professional (*n* = 29), state (*n* = 7), regional (*n* = 13), and district (*n* = 14) league coaches. The mean age of the coaches was 40.86 years (SD = 10.39); they had been coaching, on average, for 16.14 years (SD = 10.14). They represented 63 different teams, from 59 clubs, covering a variety of different team sports, including basketball (*n* = 24), volleyball (*n* = 20), ice hockey (*n* = 11), handball (*n* = 5), and field hockey (*n* = 3).

### Measures

#### Athlete Leadership Functions

The items for the four athlete leadership functions (task-oriented, relations-oriented, change-oriented, and external) were derived from Yukl (2012) and Kogler Hill’s (2016) conceptualization of these four functions – that is, the authors compiled descriptions that captured the essence of each of the four functions. To do so, the authors developed 20-item statements. They represent the 15 sub-dimensions from Yukl’s (2012) taxonomy of leadership as well as five additional items based on the existent athlete leadership literature. In order to establish content validity, six sport psychology experts with a background in leadership and group dynamics were asked to independently rate the degree to which each description matched each of the 20 leadership dimensions, which satisfied Lynn’s (1986) recommendation of at least five judges to avoid against chance agreement. The expert judges were asked to rate the degree to which each item matched each of the four athlete leadership functions. To reduce rating bias, the expert judges were provided with the items but were not told which items linked to the four athlete leadership functions. The expert judges rated each item on a five-point Likert scale from 1 (poor match) to 5 (excellent match) based on the suggestions from Dunn et al. (1999).

Decisions on whether to retain or revise items were based on Aiken’s (1985) validity (*V*) index and the qualitative feedback from the expert judges. The *V* coefficients were compared to Aiken’s table, and coefficients larger than 0.79 were statistically significant at the 0.05 level. Nineteen out of 20 sub-dimensions showed a significant match (*V* > 0.79, *p* < 0.029). Only one item description, *establishing structure*, indicated a non-significant match (*V* = 0.42, *p* > 0.05). *Establishing structure* was then modified based on the feedback from the experts. Lastly, the 20 sub-dimensions were put into each of their respective four athlete leadership functions (task-oriented, relations-oriented, change-oriented, and external) to create a composite description of that particular function. The experts concluded that the descriptions presented in Table 1 reflected the respective dimensions. For example, the description for social-oriented leadership read: “This person promotes teamwork and engagement amongst team members. He/she provides feedback, advice and/or mentoring in order to help individual team members develop. He/she fosters a constructive way of dealing with conflicts that may arise to maximize the team’s effectiveness. He/she recognizes and praises team members for good performance. He/she shows concern for individual members, provides support and is trusted by them. He/she sets an example for teammates to follow that is consistent with the values of the team.” The participants rated each of their teammates (athletes) or players (coaches) on each of the four athlete leadership functions using a seven-point Likert scale ranging from 1 (strongly disagree) to 7 (strongly agree). The original model was produced in English; therefore, the model was first translated to German by following a back-translation procedure (Brislin, 1979). In order to uphold meaning for the sports context, we engaged in three back-translation iterations, including a version from a professional translator and two versions from sport psychology experts. The outcome was discussed among a group of four sport psychology experts until a consensus was reached. Based on the German version of our extended model, we derived composite items serving as descriptions for each of the four leadership dimensions. This approach builds on earlier paradigms to identify and evaluate athlete leadership (Loughead and Hardy, 2005; Eys et al., 2007; Fransen et al., 2015).

**TABLE 1:** A four-dimensional model for the study of athlete leadership.

	Dimension	Description
Task-oriented functions	Clarifying goals	Helps the team focus on its goals
	Establishing structure	Clarifies and coordinates team activities, determines the steps and resources necessary to accomplish these activities
	Decision-making	Identifies team-related problems and facilitates decisions to resolve these
	Maintaining standards of performance	Makes sure the team’s and/or team members’ performance are meeting or exceeding expectations
	Training	Helps team members develop their skills and tactics
Relations-oriented functions	Personal development Managing conflict	Provides feedback, advice, and/or mentoring in order to help individual team members develop Fosters a constructive way of dealing with conflicts that may arise to maximize the team’s effectiveness
	Promoting teamwork	Promotes teamwork and engagement among team members
	Recognizing	Recognizes and praises team members for good performance
	Individual support	Shows concern for individual members, provides support, and is trusted by them
	Role modeling	Sets an example that is consistent with the values of the team for teammates to follow
	Empowering	Considers the suggestions of teammates and involves them in important decisions
Change-oriented functions	Inspirational motivation	Promotes a positive vision concerning the future of the team
	Intellectual stimulation	Challenges team members to think about problems in new ways
	Advocating change	Explains why change is desirable for the team
	Fostering collective learning	Encourages learning between team members to help the team develop
External-oriented functions	Networking	Develops and/or maintains favorable relationships with others outside the team who can provide useful information or assistance
	Representing team	Represents the team’s interests in meetings with coaching staff, administrators, or key stakeholders
	External monitoring	Observes the environment to identify opportunities for the team or to protect it from distractions and unnecessary demands
	Information gathering	Assesses information about the team’s performance and shares relevant information with the team

#### Perceived Leadership Effectiveness

To assess perceived leadership effectiveness, we used three items for our athlete participants and two items for our coach participants. These items were adapted from van Knippenberg and van Knippenberg (2005) and had been translated to German (Van Quaquebeke et al., 2011). One item had not been translated before and needed to be subjected to the back-translation process as described above. Because we were also assessing informal leadership and the original items came from a business context, we slightly adapted the items by replacing specific leadership terminologies with more general ones. The items included: “This person is very effective as a leader,” “He or she is a good leader,” and “This person motivates me to exert myself on behalf of the team.” The participants indicated their agreement on statements of leader effectiveness for their fellow team members using a seven-point scale ranging from 1 (totally disagree) to 7 (totally agree). We computed a composite score of perceived leadership effectiveness for every rated player by averaging the responses to each item. An analysis of reliability showed a good level of internal consistency (Cronbach’s alpha = 0.88). Intraclass correlations (ICC) indicated that, for players, 22% of total variance in perceived athlete leadership effectiveness is attributable to individuals (ICC_1_ = 0.22, ICC_2_ = 0.79). For coaches, we excluded one item because it did not fit the coaches’ perspective (“This person motivates me to exert myself on behalf of the team”). Thus, the composite score of the averaged perceived leadership effectiveness consisted of only two items. For the reliability analysis, we used Spearman–Brown statistic (*R* = 0.93, ICC_1_ = 0.19, ICC_2_ = 0.79), which provides a better estimate for two-item scales than coefficient alpha (Eisinga et al., 2013).

### Procedure

Approval for the study was obtained from the first author’s university research ethics commission^1^. An email detailing the nature of the study, including a link to the online survey, was sent to sport associations, clubs, coaches, and athletes. In addition, we also approached, in person, league organizers and individual clubs to recruit participants at tournaments and team practices. Data collection occurred electronically, both online and offline, using Qualtrics software. Due to the geographical location of the lead researcher, most athletes completed the survey offline (60.25%), while most coaches completed the survey online (87.3%). The questionnaire first asked the participants to list all players in their current team. Every participant then rated every player in their team in terms of perceived athlete leadership effectiveness and athlete leadership functions.

### Data Analysis

To test our hypotheses, the data were analyzed using multilevel modeling to account for the nested structure of the data. This controls for the dependency within the data, which originates from the same sources that provided multiple ratings (ratings nested within players). The analysis was conducted with R version 3.52 (R Core Team, 2019). Our model consisted of two levels, which distinguished between within-individual variance (level 1) and between-individuals variance (level 2). In a multilevel analysis, an unconditional model (null model) serves as a starting point for further analysis (Raudenbush and Bryk, 2002; Nezlek, 2011). This model includes a random intercept and excludes all predictors. In general, the unconditional model serves different functions. First, it assesses the need for multilevel modeling by indicating whether there is significant variation in the intercept across individuals. Second, it shows the distribution of total variability across different levels. Lastly, it provides a basis for evaluating the predictive improvement of additional models. For our analyses, we built our models incrementally by first adding the three predictors of social-, task- and external-oriented leadership to the unconditional model. This represented the original three-dimensional model of athlete leadership that has been examined in previous research (3D model). Subsequently, we added change-oriented leadership to the previous model, representing the four-dimensional model (4D model). Extending the three-dimensional model (3D model) with change-oriented leadership enables us to test both sets of hypotheses. First, controlling for all predictors of the three-dimensional model (3D model), a significant predictor of change-oriented leadership would support hypotheses 1a (players) and 1b (coaches), showing that change-oriented leadership explains unique variance. Second, a comparison of both models (3D and 4D model), indicating a significant better model fit for the four-dimensional model, would support hypothesis 2a (players) and 2b (coaches), showing that the inclusion of change-oriented leadership leads to less unexplained observations.

Furthermore, because perceived athlete leadership effectiveness was assessed with only two out of three items for coaches, we included an additional test of all three models with the same items for players (retest). This served the purpose of providing a set of models which are comparable to the coach sample. All models were evaluated by assessing individual predictors as well as comparing improvements in model fit. For all our multilevel analyses, we used maximum likelihood estimation. The predictor values were group-mean-centered as we targeted relationships on the first level of analysis (Enders and Tofighi, 2007).

## Results

For players and coaches, means and standard deviations were calculated. In the player sample, the average ratings were *M* = 3.92 (SD = 1.72) for social-oriented leadership, *M* = 3.74 (SD = 1.77) for change-oriented leadership, *M* = 3.64 (SD = 1.77) for task-oriented leadership, and *M* = 3.36 (SD = 1.88) for external-oriented leadership. In the coach sample, the average ratings were *M* = 3.78 (SD = 1.84) for social-oriented leadership, *M* = 3.58 (SD = 1.85) for change-oriented leadership, *M* = 3.48 (SD = 1.81) for task-oriented leadership, and *M* = 3.29 (SD = 1.83) for external-oriented leadership. The average ratings on perceived leadership effectiveness were *M* = 3.8 (SD = 1.66) in the player sample and *M* = 3.61 (SD = 1.77) in the coach sample. A summary of bivariate correlations among all variables is presented in Table 2. To test our hypotheses, we conducted multilevel modeling on the four different models that are presented below. The results begin with a test of the null model, followed by the 3D model and the 4D model. Lastly, we included a retest model for the player sample with two athlete leadership effectiveness items.

**TABLE 2:** Correlations for all study variables.

	Players					Coaches				
	1	2	3	4	5	1	2	3	4	5
PLE		0.75	0.76	0.62	0.76		0.75	0.80	0.61	0.74
Social	0.75		0.72	0.59	0.67	0.75		0.76	0.66	0.74
Task	0.71	0.75		0.64	0.70	0.77	0.71		0.63	0.77
External	0.60	0.64	0.72		0.61	0.52	0.56	0.61		0.64
Change	0.67	0.69	0.78	0.69		0.77	0.76	0.71	0.59	

*For players and coaches, coefficients above the diagonal are within-person level 1 correlations (number of observations for players = 2125; for coaches = 989). Coefficients below the diagonal are between-person level 2 correlations. PLE refers to perceived leadership effectiveness. Social, task, external and change refer to the respective athlete leadership function.*

### Null Model

A comparison of our random intercept model (null model) to a baseline model with a fixed intercept showed that the intercepts vary significantly across individuals for players, SD = 0.78 (95% CI: 0.68, 0.90), X^2^(1) = 271.98, *p* < 0.001, as well as for coaches, *SD* = 0.75 (95% CI: 0.61, 0.96), X^2^(1) = 110.05, *p* < 0.001. Thus, the intercept is significantly different for the participants in terms of our outcome variable, which justifies the use of multilevel modeling.

### 3D Model

Next, we added all predictors of the three-dimensional model of athlete leadership in one block. This model served as a reference model to test our hypotheses. In accordance with a meta-analysis from the organizational literature, which showed that social-oriented leadership, in comparison to task-oriented leadership, had the strongest relation with leadership outcomes (Judge et al., 2004), our athlete leadership functions were added to the model in the following order: social-oriented leadership, task-oriented leadership, and external-oriented leadership. For players (H1a), all three predictors significantly predicted perceived leadership effectiveness (social, β = 0.41, *p* < 0.001; task, β = 0.39, *p* < 0.001; external, β = 0.14, *p* < 0.001). In order to evaluate the model fit and enable comparisons, we used Schwarz’s Bayesian criterion (BIC) (Field et al., 2012). BIC is more conservative than other common goodness-of-fit measures when the sample size is large and the number of parameters is small. Furthermore, there is no objective reference for what constitutes small and large values; however, BIC allows for comparisons of models predicting the same outcome variable, with smaller values representing a better model fit (for an overview of the results, see Table 3). In comparison to the unconditional model, the model fit improved significantly from the null model (BIC = 7,920.51) to the 3D model (BIC = 5,727.62), X^2^(1) = 2,232.85, *p* < 0.001. Adding two-way interaction terms between any of the three dimensions did not lead to a better model fit. In particular, for social- and task-oriented leadership, BIC = 5,735.18, X^2^(1) = 0.10, *p* = 0.75; for social and external-oriented leadership, BIC = 5,732.60, X^2^(1) = 2.68, *p* = 0.10; and for task and external-oriented leadership, BIC = 5,735.11, X^2^(1) = 0.18, *p* = 0.67.

**TABLE 3:** Multilevel regression models: estimates and fit (athletes).

	Null model	3D model	3D model^1^	4D model	4D model^1^
	B [CI] (SE)	B [CI] (SE)	B [CI] (SE)	B [CI] (SE)	B [CI] (SE)
Fixed effects					
Intercept (γ00)	3.85 [3.71, 3.99] (0.07)**	3.86 [3.72, 4] (0.07)**	3.66 [3.52, 3.8] (0.07)**	3.86 [3.72, 4] (0.07)**	3.67 [3.53, 3.8] (0.07)**
Social		0.41 [0.37, 0.45] (0.02)**	0.38 [0.34, 0.42] (0.02)**	0.31 [0.28, 0.35] (0.02)**	0.29 [0.25, 0.33] (0.02)**
Task		0.39 [0.35, 0.43] (0.02)**	0.44 [0.39, 0.48] (0.02)**	0.28 [0.24, 0.32] (0.02)**	0.32 [0.28, 0.37] (0.02)**
External		0.14 [0.11, 0.17] (0.02)**	0.17 [0.14, 0.21] (0.02)**	0.07 [0.04, 0.11] (0.02)**	0.11 [0.07, 0.15] (0.2)**
Change				0.33 [0.30, 0.37] (0.02)**	0.31 [0.27, 0.35] (0.02)**
Random effects					
Intercept	0.78 [0.68, 0.9]	0.86 [0.76, 0.97]	0.85 [0.76, 0.96]	0.86 [0.77, 0.97]	0.86 [0.76, 0.96]
Model fit					
BIC	7937.49	5727.62	6193.14	5446.47	6006.8

The predictor variables are group-mean-centered. The confidence intervals (95%) are inside the square parentheses. The standard errors are inside the round parentheses. The null model represents the unconditional model. The 3D model included the predictors of social-, task, and external-oriented leadership. The 3D model^1^ represents a retest of the 3D model, with a reduced number of items. The 4D model included the predictors of social-, task-, external- and change-oriented leadership. The 4D model^1^ represents a retest of the 4D model, with a reduced number of items. **p < 0.001.

Similarly, we tested the three-dimensional model for coaches. All three predictors significantly predicted perceived leadership effectiveness (social-oriented leadership, β = 0.34, *p* < 0.001; task-oriented leadership, β = 0.52, *p* < 0.001; and external-oriented leadership, β = 0.08, *p* < 0.01). The model fit improved from the null model (BIC = 3,844.01) to the 3D model (BIC = 2,782.16), X^2^(1) = 1,082.55, *p* < 0.001, in comparison to the unconditional model. A two-way interaction term between any of the three athlete leadership dimensions did not further improve the model fit, specifically, for social- and task-oriented leadership, BIC = 2,789.0, X^2^(1) = 0.05, *p* = 0.82; for social- and external-oriented leadership, BIC = 2,788.7, X^2^(1) = 0.35, *p* = 0.55; and for task- and external-oriented leadership, BIC = 2,788.65, X^2^(1) = 0.4, *p* = 0.53.

### 4D Model

To test hypothesis 1a and 1b, we extended the 3D model by adding change-oriented leadership (4D model). For players (H1a), all four predictors significantly predicted perceived leadership effectiveness (social-oriented leadership, β = 0.31, *p* < 0.001; task-oriented leadership, β = 0.28, *p* < 0.001; external-oriented leadership, β = 0.07, *p* < 0.001; and change-oriented leadership, β = 0.33, *p* < 0.001). Thus, we reject the null hypothesis for H1a. The model fit for the 4D model (BIC = 5,446.47) improved in comparison to the 3D model (BIC = 5,727.62), X^2^(1) = 288.82, *p* < 0.001. Thus, we reject the null hypothesis for H2a. Adding two-way interaction terms between change-oriented leadership and any of the three other leadership dimensions (i.e., social-, task-, and external-oriented leadership) did not lead to a better model fit. In particular, for change- and social-oriented leadership, BIC = 5,452.46, X^2^(1) = 1.67, *p* = 0.20; for change- and task-oriented leadership, BIC = 5,453.98, X^2^(1) = 0.15, *p* = 0.70; and for change- and external-oriented leadership, BIC = 5,452.84, X^2^(1) = 1.29, *p* = 0.26. For coaches (H2B), social-, task-, and change-oriented leadership significantly predicted perceived leadership effectiveness (social-oriented leadership, β = 0.28, *p* < 0.001; task-oriented leadership, β = 0.43, *p* < 0.001; and change-oriented leadership, β = 0.20, *p* < 0.001). Thus, we reject the null hypothesis for H1b. However, the predictor of external-oriented leadership was not significant (β = 0.05, *p* = 0.066). The 4D model (BIC = 2,752.7) in comparison to 3D model (BIC = 2,782.16) showed a better model, X^2^(1) = 36.36, *p* < 0.001. Thus, we reject the null hypothesis for H2b. The model fit was not improved by adding two-way interaction terms between change-oriented leadership and any of the three other leadership dimensions. For change- and social-oriented leadership, BIC = 2,759.59, X^2^(1) = 0.009, *p* = 0.92; for change- and task-oriented leadership, BIC = 2,758.91, X^2^(1) = 0.69, *p* = 0.41; and for change- and external-oriented leadership, BIC = 2,759.11, X^2^(1) = 0.48, *p* = 0.49. The results for the main models are presented in Table 3 for players and in Table 4 for coaches.

**TABLE 4:** Multilevel regression models: estimates and fit (coaches).

	Null model	3D model	4D model
	B [CI] (SE)	B [CI] (SE)	B [CI] (SE)
Fixed effects			
Intercept (γ00)	3.64 [3.43, 3.86] (0.11)**	3.65 [3.43, 3.86] (0.11)**	3.65 [3.43, 3.86] (0.11)**
Social		0.34 [0.28, 0.4] (0.03)**	0.28 [0.22, 0.34] (0.03)**
Task		0.52 [0.46, 0.58] (0.03)**	0.43 [0.37,0.5] (0.03)**
External		0.08 [0.03, 0.13] (0.03)*	0.05 [0, 0.1] (0.03)
Change			0.20 [0.13, 0.26] (0.03)**
Random effects			
Intercept	0.76 [0.61, 0.96]	0.84 [0.69, 1]	0.84 [0.7, 1.01]
Model fit			
BIC	3844.01	2782.16	2752.70

The predictor variables are group-mean-centered. The confidence intervals (95%) are inside the square parentheses. The standard errors are inside the round parentheses. The null model represents the unconditional model. The 3D model included the predictors of social-, task-, and external-oriented leadership. The 4D model included the predictors of social-, task-, external-, and change-oriented leadership. *p < 0.01, **p < 0.001.

For both models and samples, we tested for multilevel analysis assumptions for parametric data (Field et al., 2012). For the coach sample, normality, linearity, and homoscedasticity were inspected visually and met the requirements. Multicollinearity was tested by computing the variance inflation factor (VIF) and tolerance statistics and these indicated no violations (3D model, social-oriented leadership, tolerance = 0.37, VIF = 2.69; task-oriented leadership, tolerance = 0.39, VIF = 2.56; external-oriented leadership, tolerance = 0.53, VIF = 1.9; 4D model, social-oriented leadership, tolerance = 0.34, VIF = 2.95; task-oriented leadership, tolerance = 0.32, VIF = 3.15; external-oriented leadership, tolerance = 0.5, VIF = 1.99; change-oriented leadership, tolerance = 0.33, VIF = 3.02). For the player sample, the assumptions of normality, linearity, and homoscedasticity were equally inspected and met the requirements. The VIF and tolerance statistics likewise indicated no violations of multicollinearity (3D model, social-oriented leadership, tolerance = 0.46, VIF = 2.19; task-oriented leadership, tolerance = 0.42, VIF = 2.4; external-oriented leadership, tolerance = 0.56, VIF = 1.8; 4D model, social-oriented leadership, tolerance = 0.42, VIF = 2.4; task-oriented leadership, tolerance = 0.37, VIF = 2.7; external-oriented leadership, tolerance = 0.53, VIF = 1.9; change-oriented leadership, tolerance = 0.42, VIF = 2.37). There were no missing values in our data.

### Retest

To account for the missing item in the coaches’ data, we tested all three models with the players’ data for the same two items that the coaches completed. All retested models are indicated with a superscript numerator. The comparison of the null model^1^ to the fixed intercept baseline model showed that the intercepts varied significantly across individuals for players, justifying the use of multilevel modeling, X^2^(1) = 224.23, *p* < 0.001. Intraclass correlations indicated that, for players, 19% (ICC1 = 0.19, ICC2 = 0.76) of total variance in perceived leadership effectiveness was attributable to individuals (between-individuals variance). The retest of the three-dimensional model, 3D model^1^, showed that all three predictors significantly predicted perceived leadership effectiveness (social-oriented leadership, β = 0.38, *p* < 0.001; task-oriented leadership, β = 0.44, *p* < 0.001; and external*-*oriented leadership, β = 0.17, *p* < 0.001). In comparison to the unconditional model, the model fit improved from the null model^1^ (BIC = 8,200.80) to the 3D model^1^ (BIC = 6,193.14), X^2^(1) = 2,030.64, *p* < 0.001. For the 4D model^1^, all four predictors significantly predicted perceived leadership effectiveness (social-oriented leadership, β = 0.29, *p* < 0.001; task-oriented leadership, β = 0.32, *p* < 0.001; external-oriented leadership, β = 0.11, *p* < 0.001; and change-oriented leadership, β = 0.31, *p* < 0.001). The model fit for the 4D model^1^ improved in comparison to the 3D model^1^ (BIC = 6,193.14) and to the 4D model^1^ (BIC = 6,006.8), X^2^(1) = 194.00, *p* < 0.001. For these models, the assumptions of normality, linearity, and homoscedasticity were also inspected visually and met the requirements. The VIF and tolerance statistics indicated no violations of multicollinearity as indicated by the values from the prior test for the player sample.

## Discussion

The results for players and coaches support the inclusion of change-oriented leadership as a fourth dimension within the athlete leadership taxonomy. Specifically, for both players and coaches samples, controlling for task-, social-, and external-oriented leadership, change-oriented leadership significantly predicted athlete leadership effectiveness. Furthermore, the inclusion of change-oriented leadership increased the model fit in comparison to the three-dimensional model consisting of task-, social-, and external-oriented leadership. These findings lend support for the use of a four-dimensional over a three-dimensional model in future research.

In the following section, we are looking at each predictor individually. We have structured the results by the numeric values of the individual predictors. Statistically, there is no difference for change-, social- and task-oriented leadership when considering a 95% confidence interval for players. For coaches, there is a difference between the confidence intervals of task leadership and both dimensions of social- and change-oriented leadership. External-oriented leadership is statistically lower than all other dimensions for both samples (i.e., players and coaches). Specifically, within the four-dimensional model, change-oriented leadership was shown to significantly predict perceived leadership effectiveness (β = 0.33) for players. This outcome corroborates the findings of a meta-analysis conducted by Judge and Piccolo (2004), indicating a positive relationship between transformational leadership (a form of change-oriented leadership) and leader effectiveness. The second largest predictor in our player sample was social-oriented leadership (β = 0.31), followed by task-oriented leadership (β = 0.28). The order of these two functions of athlete leadership supports previous findings that place social-oriented leadership above task-oriented leadership (Judge et al., 2004). Judge and colleagues’ meta-analysis found moderately strong relationships between consideration – a form of social-oriented leadership ($\hat{\rho }$ = 0.48) – and initiating structure – a form of task-oriented leadership ($\hat{\rho }$ = 0.29) – with leadership outcomes. In the present study, external-oriented leadership was the fourth largest predictor of leadership effectiveness (β = 0.07). This finding is similar to a previous athlete leadership research (Fransen et al., 2014), where external-oriented leadership ranked as the least important in comparison to task-oriented, social-oriented, and motivational athlete leadership functions.

For coaches, the ranking of the four athlete leadership functions is slightly different from those of our player sample. The largest predictor of perceived athlete leader effectiveness was task-oriented leadership (β = 0.43), followed by social-oriented leadership (β = 0.28), and change-oriented leadership (β = 0.20). A significant predictor of change-oriented leadership supports previous research which showed that adolescent players who used transformational leadership behaviors were seen as more effective athlete leaders by their coaches, including higher ratings of peer satisfaction with leadership as well having higher effort-enhancing skills (Zacharatos et al., 2000). External-oriented leadership was not shown to predict athlete leader effectiveness in our sample of coaches. However, it should be noted that external-oriented leadership was close to being significant. Considering the predictor weights, our results suggest that coaches appear to put particular emphasis on efforts toward goal attainment and coordination (i.e., task orientation). Change-oriented leadership was shown to be less influential for coaches than for players, which could be due to its nature of challenging the *status quo*, which implies the pursuit of “a future that is different from today” (van Knippenberg and Sitkin, 2013, 47). In more general terms, leaders have shown to be pillars of continuity and stability as well as important agents of change (van Knippenberg et al., 2008; Rast et al., 2016). In that light, task leadership could be understood as leadership functions that provide stability or, at least, do not undermine it. As such, coaches might see those as effective athlete leaders who support them by providing stability within the team. In fact, in a qualitative study investigating coaches’ perceptions of athlete leadership, the coaches reported that one of their main requirements for their athlete leaders was to follow their instructions (Bucci et al., 2012). Similarly, they expected athlete leaders to promote a team culture that was based on the coaching staff – that is, coaches prefer athlete leaders to be an extension of the coaching staff. Therefore, it is not surprising that change-oriented leadership ranked lower than task-oriented leadership. It was still a significant predictor of perceived athlete leadership effectiveness. However, Bucci et al. (2012) also found that one of the coaches reported that there is value in athlete leaders providing different types of leadership. He had selected a leadership group with complementary skills in order to extend the leadership capacities of the team. Specifically, he selected two types of athlete leaders that would either support or reject normative behavior. Taken together, the results suggest that athletes and coaches seem to prioritize different dimensions of what constitutes athlete leadership effectiveness.

In general, our results suggest that change-oriented leadership represents an important extension of the previous conceptualization of athlete leadership as a function of three leadership dimensions. The inclusion of change-oriented leadership raises several questions that need to be addressed in future research. We see five key issues that require further attention: first, the investigation of change orientation as a critical dimension of athlete leadership. The present study has shown that change-oriented leadership plays an important role for the behavioral skillset of athlete leaders. For instance, future studies could examine athlete leadership as an important resource for creating and communicating visions for team development as well as for the general process of change management. This could be particularly important for transition periods, such as changes within the coaching staff, as well as transitions between seasons.

Second, the investigation of differences between coaches’ and players’ expectations toward athlete leadership appears to be a fruitful area of research. Previous research has demonstrated that athletes and coaches provide different types of leadership (Loughead and Hardy, 2005). These differences possibly imply that coaches also have different expectations toward athlete leaders to engage primarily in support of task- and social-oriented leadership, while athletes, in contrast, seem to be particularly responsive to change-oriented leadership behavior from other team members. For instance, coaches might view athlete leadership as a means to coordinate team efforts toward the attainment of season goals, while athlete leaders might see a need for change and work toward a different future. In that light, qualitative research could provide more insight into the dynamics between coaches’ and athletes’ perspectives on athlete leadership.

Third, future research should examine the four-dimensional taxonomy on a sub-dimensional level. In line with previous athlete leadership research (Eys et al., 2007; Duguay et al., 2019a; Fransen et al., 2019), we decided to first examine change orientation at the dimensional level. While this level of analysis is similar to previous research, applied research should benefit from further differentiation between the gross leadership dimensions. For instance, athlete leadership development research has used specific behavioral dimensions in the training of athlete leadership (Duguay et al., 2016). An expansion of the existing vocabulary of leadership functions could enable future research to cover a wide range of behaviors as well as to address more specific research questions.

Fourth, we would like to emphasize that a focus on behavior constitutes only one part of understanding athlete leadership as a group phenomenon. The assessment of leadership behavior singles out the individual and disregards the social context. This is particularly relevant for athlete leadership from a shared leadership perspective, which defines the construct as “an emergent team phenomenon” (Carson et al., 2007). Put differently, at the team level, athlete leadership can be seen as the product of “dynamic interactions among lower-level elements” (Kozlowski and Klein, 2000, 15). Thus, the results of the present study should be considered within the lower levels of a multi-level phenomenon. In that light, future research should address antecedents and processes for the emergence of all dimensions, including change-oriented leadership at the team level.

The fifth area of research concerns the reconciliation of motivational leadership with the four-dimensional athlete leadership taxonomy. A primary goal of this study was to provide a structure for future athlete leadership research. Just like task-, social-, and external-oriented leadership, change-oriented leadership originated from social and organizational psychology. By considering the same four meta-categories, future athlete leadership research should be able to reconnect findings to interdisciplinary leadership research. While every discipline has to attend to context-specific characteristics, there is much common ground (e.g., Mullen and Copper, 1994). Therefore, on a fundamental level, athlete leadership research should be able to relate to empirical findings in organizational and social psychology. As mentioned earlier, the construct of motivational leadership bares commonalities with the meta-category of change orientation. At its core, motivational leadership serves the “encouragement of teammates to go the extra mile” and steering of “all emotions […] in the right direction” (Fransen et al., 2014). Similar words have been used to describe the effects of transformational and charismatic leadership. For instance, “leaders cause followers (…) to perform above and beyond the call of duty” and increase the “emotional and motivational arousal of the followers” (Shamir et al., 1993, 577). However, comparisons beyond wording are not possible since the concept did not stem from theory but from field research. Moreover, transformational leadership is a multifaceted construct that does not solely build on change-oriented dimensions (e.g., individualized consideration). Nevertheless, motivational leadership has spurred numerous studies and been shown to correlate with team functioning (Fransen et al., 2017). Hence, future research should seek to reconcile motivational leadership within the four-dimensional framework proposed in this study. An investigation of the four-dimensional taxonomy on a sub-dimensional level could be a next step in that direction.

Moreover, our results have implications for applied practice. By definition, change-oriented functions aim to successfully adapt to change in the environment. For that, leaders can act as important drivers of change by communicating a vision and advocating the necessity of change (Herold et al., 2008). So far, athlete leadership research has mostly neglected this side of athlete leadership. Therefore, teams that struggle with changes in the environment might benefit from athlete leadership development as a pillar of successful change management. Coaches could consider close cooperation with a leadership group to steer it through critical team changes. Recently, there has been a rise of interest in athlete leadership development programs (Loughead et al., 2020). Considering that coaches might understand the roles of athlete leaders differently than team members, practitioners might consider integrating the coach into the athlete leadership development process. By that, they could help the team to find a dynamic that allows athlete leaders to cover all aspects of leadership while reconciling these roles with the coaches’ expectations.

The limitations of the current study are twofold. First, we chose to measure each of the four meta-categories instead of the 20 sub-dimensions. With regards to external orientation, which was not found to be a significant predictor of athlete leadership effectiveness for coaches, the use of composite items could have been marginalizing. The sub-dimension of *representing team*, for instance, refers to leadership behavior that mediates between the team and its immediate environment (i.e., coaching staff, management). Whereas the sub-dimension of *networking* could be seen as a form of mediation, which goes beyond the team’s immediate environment (e.g., team consultants). In this case, the use of single-item composites might have led to a loss of information that did not differentiate between more and less influential sub-dimensions of external orientation. Hence, the fact that external-oriented leadership did not significantly predict leadership effectiveness for coaches has to be interpreted cautiously. However, an analysis on the level of meta-categories has been utilized in previous athlete leadership research (e.g., Eys et al., 2007; Duguay et al., 2019a; Fransen et al., 2019). Moreover, it was necessary to first investigate the significance of change orientation with the realm of athlete leadership. This is why we chose this level of analysis as an important first step in the current study. Another limitation in using single-item composites is that we could not provide a measure of reliability. However, a similar progression has been shown in organizational leadership, where Yukl’s (2012) taxonomy established a framework that spawned several research studies, such as a shared leadership questionnaire (Grille and Kauffeld, 2015). A next step could likewise be the development of a psychometrically sound questionnaire for shared athlete leadership. Second, since we did not target any interactive team sport in particular, our sample was rather heterogeneous. This limited us in terms of exploratory analysis, such as the nuances of one sport on the relationship between behavior and perceived leader effectiveness. However, the use of different sports, leagues, and age groups added to the level of generalizability of our results.

In conclusion, we view change-oriented leadership as an important and relevant dimension for the study of athlete leadership. In organizational leadership research, change-oriented leadership has long been recognized as a fundamental aspect of this construct (Avolio and Yammarino, 2013). Our findings support its significance within athlete leadership research. For that, the existence of an athlete leadership taxonomy helps to structure future research endeavors, highlight research gaps, and provide an overview in the complex and diverse field of athlete leadership research.

## Data Availability Statement

The datasets generated for this study can be found in the Open Science Framework (OSF) repository: https://osf.io/kn42w/?view_only=386908061ef94997aae5bc3800cdc4cb.

## Ethics Statement

The studies involving human participants were reviewed and approved by the Ethics Committee at the Technical University of Munich. Written informed consent from the participants’ legal guardian/next of kin was not required to participate in this study in accordance with the national legislation and the institutional requirements.

## Author Contributions

The original research is part of the Ph.D. thesis of CM, supervised by JB and TL. CM and TL are responsible for the conceptualization of the study as well as the development of the four-dimensional model of athlete leadership. Data collection and data analysis were conducted by CM. All the authors contributed to the writing of the manuscript and approved its final version.

## Conflict of Interest

The authors declare that the research was conducted in the absence of any commercial or financial relationships that could be construed as a potential conflict of interest.

## Footnotes

1 Ethics Commission of the Technical University of Munich.
